# The Probabilistic Convolution Tree: Efficient Exact Bayesian Inference for Faster LC-MS/MS Protein Inference

**DOI:** 10.1371/journal.pone.0091507

**Published:** 2014-03-13

**Authors:** Oliver Serang

**Affiliations:** Thermo Fisher Scientific, Bremen, Germany; Indiana University, United States of America

## Abstract

Exact Bayesian inference can sometimes be performed efficiently for special cases where a function has commutative and associative symmetry of its inputs (called “causal independence”). For this reason, it is desirable to exploit such symmetry on big data sets. Here we present a method to exploit a general form of this symmetry on probabilistic adder nodes by transforming those probabilistic adder nodes into a probabilistic convolution tree with which dynamic programming computes exact probabilities. A substantial speedup is demonstrated using an illustration example that can arise when identifying splice forms with bottom-up mass spectrometry-based proteomics. On this example, even state-of-the-art exact inference algorithms require a runtime more than exponential in the number of splice forms considered. By using the probabilistic convolution tree, we reduce the runtime to 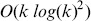 and the space to 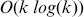 where 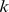 is the number of variables joined by an additive or cardinal operator. This approach, which can also be used with junction tree inference, is applicable to graphs with arbitrary dependency on counting variables or cardinalities and can be used on diverse problems and fields like forward error correcting codes, elemental decomposition, and spectral demixing. The approach also trivially generalizes to multiple dimensions.

## Introduction

In bottom-up mass spectrometry pieces of digested proteins, which are called peptides, are first matched to observed spectral evidence, and the quality of the match between the peptide and the spectrum is scored[1]–[5]. These scored peptides are then used to perform inference on the proteins, whose present-absent states are usually the variables of interest. The computational cost of inference is non-trivial: some graphs can be processed efficiently, while performing inference on other graphs can be proven to solve the NP-hard minimum set cover problem [2]. Performing efficient but accurate inference on these graphs is important for producing reliable protein inferences.

**Figure 1:**
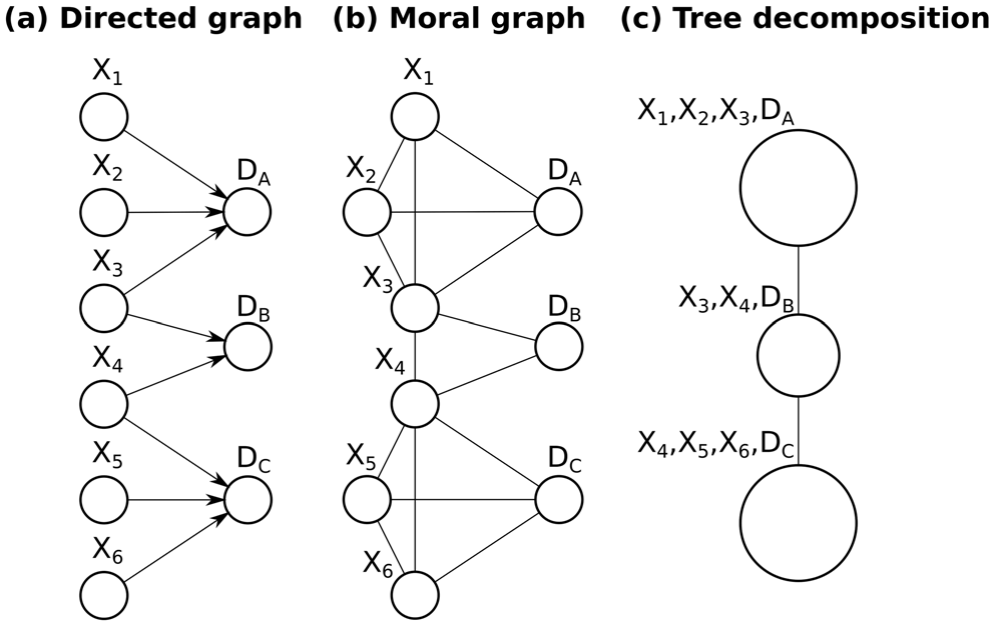
Mass spectrometry: a graphical view. (**a**) Directed edges between proteins 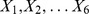 and spectral data 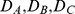 represent causal statistical dependencies with spectra that can result from peptides in the adjacent protein. For simplicity, peptide-spectrum-matches (PSMs) are denoted simply using their spectral evidence, thereby producing a bipartite graph of proteins (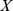) to spectral data (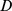). Proteins 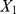, 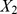, and 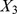 share spectral data 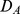, because they share peptides that were matched to spectrum 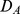. : if the score of the PSM corresponding to spectrum 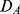 is very high, it is tempting to award a high probability to protein 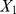; however, proteins 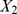 and 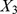 compete for this shared evidence, and thereby have a chance to reduce the probability of 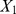. This process (called “explaining away” to describe the fact that the contribution of evidence to a single hypothesis is reduced by competing hypotheses) introduces new dependencies between all pairs of proteins sharing that evidence. (**b**) These shared spectral data introduce new non-causal dependencies between proteins with shared successors in (a). These dependencies are visualized in the undirected moral graph. When multiple proteins share spectral evidence, these undirected edges connect all pairs of predecessors, creating a clique in the moral graph 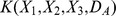. (**c**) The tree decomposition (sometimes called the “junction tree” or “clique tree”) merges the moral graph from (b) without loss of dependencies, so that inference can be performed using Pearl’s belief propagation algorithm. Belief propagation starts at the top clique, which only shares variable 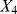 with its neighbor. Therefore, the top clique can perform inference while leaving 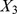 as a symbolic, unknown quantity, so that it can be used to send information from the cliques below (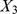 is an information bottleneck, through which the cliques below can influence the top clique). Likewise, the variables 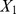 and 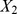 can be marginalized out before sending any relevant information to considering the middle clique. This procedure can significantly reduce the runtime by allowing inference to be performed on the cliques rather than on all nodes in the tree; however, each clique represents an inseparable multidimensional distribution over several variables, and thus the cost of processing a single clique is more than exponential in the number of variables. When many proteins share common evidence (*i.e.* share at least one peptide identified by spectral evidence), a large clique is formed in the moral graph and inference becomes intractable in the general case.

Figure 1 depicts a simple graphical view of protein identification from tandem mass spectrometry experiment. In figure 1a, the causal flow of information is described graphically: proteins are digested and then fragmented to produce observed spectral evidence. Directed edges between proteins 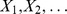 and spectral data 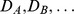 represent causal statistical dependencies: proteins are connected to the MS/MS spectra matching peptides that can be produced according to the model of the digest (*e.g.* peptide strings resulting from an *in silico* digest of protein sequences using trypsin cleavage rules with up to one missed cleavage). Note that because peptides are usually paired with spectrum in a one-to-one manner (pairing each peptide to its best-matching spectrum and pairing each spectrum with its best matching peptide to form a “PSM” or peptide-spectrum match), we simply draw the proteins producing that spectral evidence, thereby producing a bipartite graph of proteins (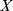) to spectral data (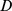). Note that this one-to-one mapping between peptides and spectra means that this bipartite graph can be viewed as a protein-peptide bipartite graph; however, rather than constrain ourselves to inference with MS/MS intensities, the data used for inference could just as easily constitute precursor MS intensities or even spectra resulting from a top-down experiment.

Proteins with shared spectral data (such as 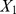, 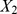, and 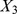, which share evidence 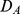, which could describe an identified “degenerate” peptide) introduce new non-causal dependencies: if the score of the PSM corresponding to spectrum 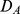 is very high, it is tempting to award a high probability to protein 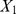; however, proteins 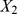 and 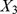 compete for this shared evidence, and thereby have a chance to reduce the probability of 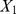. This process (called “explaining away” to describe the fact that the contribution of evidence to a single hypothesis is reduced by competing hypotheses) introduces new dependencies between all pairs of proteins sharing that evidence.

In figure 1b, we illustrate those shared protein-to-protein dependencies with the moral graph [6] (so called because “parent” nodes, which share a common child are “married by an edge”). The moral graph displays these latent dependencies between proteins, as well as the original causal dependencies from (a). Hence, when multiple proteins share spectral evidence, these undirected edges create a clique in the moral graph 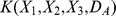. These cliques can be joined in a tree, which is known as the tree decomposition [7] (also known as the “junction tree” or “clique tree”).

In figure 1c, we show a tree decomposition formed from the moral graph in figure 1b. This tree decomposition is performed by merges nodes in the moral graph so that the resulting graph can be viewed as a tree without losing any edges (lost edges would correspond to ignored dependencies). Posterior probabilities on graphs without cycles (*i.e.* trees) can be computed by visiting each clique only twice using Pearl’s belief propagation algorithm; however, each clique represents an inseparable multidimensional distribution over several variables, and thus the cost of processing a single clique is more than exponential in the number of variables (its state space is the Cartesian product of the contained variables’ outcomes). Therefore, the runtime of junction tree inference is more than exponential in the size of the largest clique (the “tree width” of the graph is the size of the largest clique minus one).

For this reason, when many proteins share common evidence (*i.e.* in tandem mass spectrometry, at least one shared peptide matching spectra), a joint dependency between all of those proteins is created, and the large resulting clique formed in the moral graph can make inference intractable in the general case. Without modifying it after tree decomposition, exact inference on the graph is more than exponential (that is, it is not in 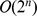) in the number of proteins joined by such evidence [8]. And because these large cliques represent a full joint distribution of dimension 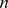, even sampling procedures like Monte Carlo and Markov chain Monte Carlo (MCMC), which have been successfully applied to protein inference[8]–[10], cannot saturate the space with samples, and are thus insufficient.

**Figure 2:**
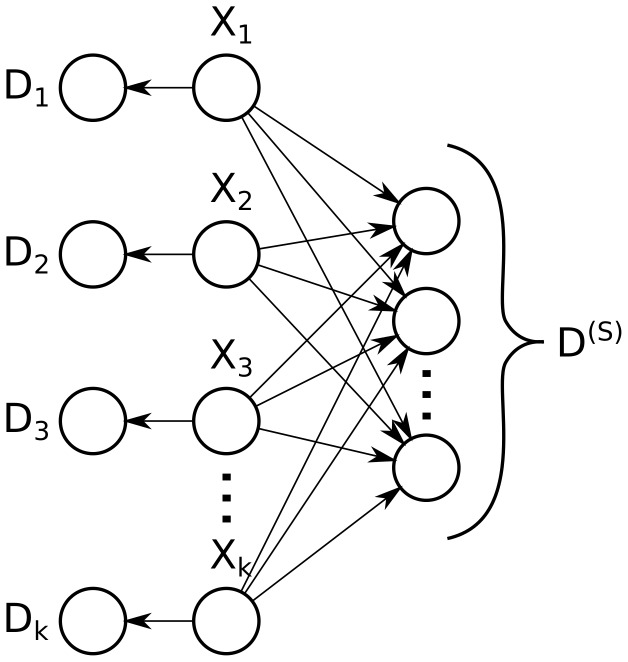
Difficult inference: mass spectrometry-based identification of splice variants. Several proteins 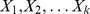 matching unique and shared peptide-level evidence. The peptide-level evidence, 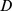 is partitioned into unique peptide-level evidence (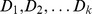) as well as a collection of shared peptide level evidence shared by all proteins (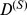). Graphs of this form are typical when searching mass spectra against protein databases containing substantial redundancy (*e.g.* databases with many splice variants or close homologs), because these types of proteins share core similarities but also have unique regions that distinguish them from one another. Inference on this type of graph cannot be performed efficiently through protein clustering, protein pruning, or junction tree decomposition; to date, exact Bayesian protein inference on such splice variant graphs has only been performed in super-exponential time.

In particular, Bayesian networks similar to the one shown in figure 2 can occur in mass spectrometry-based proteomics when attempting to identify homologous (or, more generally, proteins with sequence similarity). Specifically, these challenges occur in practice when attempting to distinguish between antibodies and other closely related splice variants [11], searching large databases containing non-canonical variants to find aberrant gene products (*e.g.* from samples of cancerous tissue), and in proteomic studies of organisms for which little or low-quality genomic information is available [12]. The entirety of spectral data for this graph, 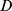, is partitioned into two categories: First, all proteins 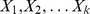 in the weakly connected subgraph are adjacent to a collection of shared evidence (*e.g.* degenerate peptides matching observed spectra). These shared data, denoted 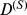, produce a large clique in the moralized graph, which indicates that computing and passing the messages will be computationally infeasible on large problems without considering the form of the conditional probability function for the shared data. Second, each protein also has any number of unique evidence, which are found only in a single protein. The evidence (*e.g.* peptides) unique to protein 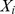 are denoted 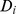.

### A Previous Model for Bayesian Protein Inference

Previous work on exact Bayesian inference for protein identification [2], [13] models every protein with an independent identically distributed prior probability 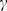 that the protein is truly in the sample, a conditional probability 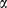 that a present protein would generate a constituent peptide, and a noise model where incorrect peptide identifications were modeled with probability 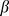. Lastly, the event that one present protein is successfully digested into one of its shared constituent peptides does not influence the event that another protein is successfully digested into the same peptide.

### Optimizations for more Efficient Protein Inference

Using this model, exact inference can sometimes be performed more efficiently using two optimizations: clustering and pruning. These two optimizations are used in conjunction with factorization, which separates and performs inference individually on weakly connected subgraphs, to perform exact inference more quickly.

The first optimization, clustering, merges together any collection of 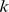 protein nodes 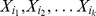 that contain identical peptide sets. Because the probability that a peptide is absent is the probability that it came from neither the noise model nor from any proteins:
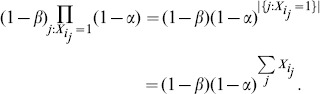



These proteins were clustered because they share *identical* peptide sets, such that all spectral evidence depending on these proteins actually depends on the number of protein present 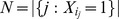 rather than on the actual set of present proteins. This is because addition is a commutative and associative operation, and so rather than enumerate the power-set of proteins, marginalization can instead be performed on the number of proteins present:
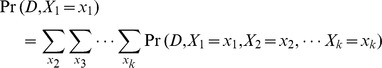





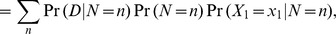
where 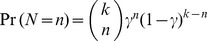 and the conditional probability for an arbitrary protein 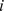 in the cluster 
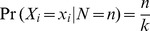
. Thus enumeration, which has a computational cost that exceeds 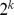, is reduced to 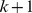 steps. However, clustering cannot be performed unless the proteins clustered are adjacent to *identical* sets; the graph in figure 2 does not have this property, because proteins are potentially distinguishable by their unique evidence 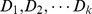.

The second optimization, pruning, exploits the fact that this type of protein-peptide emission model (sometimes referred to as a “noisy-or” model with symmetric parameters) produces independent nodes when a peptide is *not* observed (*i.e.* when a peptide has a zero probability of matching any observed spectra). Thus, these zero-score shared peptides found in 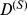 can be copied so that each protein has its own unique copy. If all shared peptides from 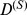 have zero scores, the resulting graph can thus be solved in linear time because all proteins are now independent. Unfortunately, as was the case with clustering, this optimization fails on figure 2 except in the rare case when *all* peptides in 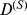 have zero scores (in practice, it is rare that all such peptides will have zero scores on a large problem). Approximations of the posterior probabilities can be computed by pruning or removing some select peptides that have nonzero scores; however, this can result in lower accuracy when searching spectra against complex protein databases, which have many more such shared peptides [3].

As noted above, the junction tree algorithm, clustering optimization, and pruning optimization do not solve the problem from figure 2 in sub-exponential time; on the contrary, the most efficient exact Bayesian algorithm that has been demonstrated for this type of splicoform graph enumerates the power-set [8]. Furthermore, clustering relies heavily on the assumptions in the original model, and requires that the protein prior 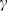 must be the same for all proteins, and the peptide emission probability 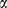 must be the same for all peptides (and the method used to duplicate pruned peptides in the supplement of [13] assumes the noise model 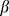 is identical for all peptides). In a similar manner, pruning is tied to the use of noisy-or peptide nodes. Likewise, other optimizations are rigidly tied to specific graph topologies. For example, inference can be performed in linear time on polytrees that exclusively use noisy-or nodes by decomposing the noisy-or nodes in an iterative fashion and performing belief propagation [6], [14]; however, the graph from figure 2 is a polytree only when there is exactly one node of shared evidence in 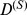 (for completeness, when there are *zero* nodes of shared evidence, the graph can also be solved efficiently by partitioning it into a collection of disjoint polytrees).

### The Need for Further Optimization of Inference

Because the optimization strategies mentioned above are limited to certain graphs or require all peptides to use identical parameters 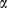 and 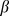, it can be difficult to use these optimization techniques to realize efficient inference using a modified or extended protein inference model: for example, the next generation of models could include arbitrary categorical priors on how many proteins are present or peptide-specific emission and noise models, which may even depend on the number of present predecessor proteins (rather than constraining that predecessors contribute in a simple multiplicative manner as modeled by noisy-or nodes). These more general priors and emission models can be used to more objectively model the process by which splice variants data are produced by mass spectrometric analysis, and thus permit inference techniques that more accurately model the manner with which proteins compete for shared peptide data. For instance, perhaps a sixth present predecessor protein does not substantially increase the peptide’s chances compared to five present proteins, or perhaps genetic or biochemical analysis has demonstrated that it is improbable for the gene in question to express more than 7 splice forms in a short period of time. Such extensions can be implemented with *probabilistic adder* nodes, which add two probabilistic quantities. As we demonstrate in this paper, these probabilistic adder nodes can be more flexible and general than symmetric noisy-or nodes (symmetric noisy-or nodes can be trivially emulated using probabilistic adder nodes, but the converse is not true). Generalizing to probabilistic adder models offers a substantial increase in model flexibility. Because they are more general than noisy-or nodes, more problems can be decomposed into a polytree of probabilistic adder nodes (*e.g.* the graph in figure 2, even when using peptide-specific emission and noise parameters).

In this paper we present a dynamic programming method for computing exact posteriors for efficiently solving the problem in figure 2 using a more flexible probabilistic adder model. The first algorithm presented performs inference in quadratic time and quadratic space, which is compatible with a peptide-specific emission model, with a peptide-specific noise model, and with non-identical protein priors. We then generalize the algorithm we use to reveal it is an instance of the transform proposed by Heckerman[15]–[17].

Lastly, we propose the probabilistic convolution tree, a data structure which uses dynamic programming to perform exact inference on polytrees of probabilistic adder nodes in 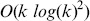 time and 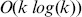 space, and compare performance of the three algorithms, power-set enumeration, quadratic dynamic programming, and the probabilistic convolution tree, on problems of the form shown in figure 2. The probabilistic convolution tree is applicable to more general graphs, can be combined with junction tree inference, and can even used to efficiently compute posteriors when using an arbitrary categorical prior on the number of present variables.

## Materials and Methods

Using graphs of the type shown in figure 2, we compare the three exact inference methods described in this paper that can be applied with peptide-specific probabilities: power-set enumeration, quadratic dynamic programming, and the proposed probabilistic convolution tree approach.

For each problem of a given size 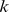, each protein is adjacent to a unique peptide and all proteins are adjacent to between 1 and 9 shared peptides (chosen using uniform integers in 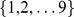). Each unique peptide 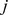 has its own 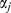 and 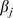 chosen uniformly 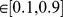 and a random probability score (*i.e.* the probability it matched an observed spectrum) chosen uniformly 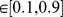. Each protein 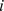 has a protein-specific prior 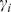 chosen uniformly 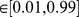 (this is useful to test the numeric stability of the algorithm, because the only division operation is based on this collection of protein priors). Lastly, the shared data 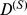 has an arbitrary categorical distribution in the number of present proteins 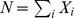 (each entry chosen randomly in 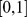 and normalized to sum to 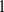).

Runtimes were compared using python implementations of each of the three algorithms on a Core i3 laptop. For all problems timed, the three methods achieve identical results (to the 8 decimal points that by default are printed out by python’s numpy package).

## Results

### The Brute-force Algorithm: Power-set Enumeration

Power-set enumeration is quite simple: the likelihood is computed for every possible joint state for all proteins 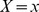.
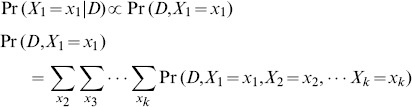





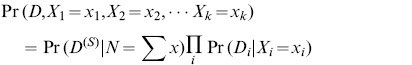



#### Illustrative example of the exponential enumeration algorithm

**Figure 3:**
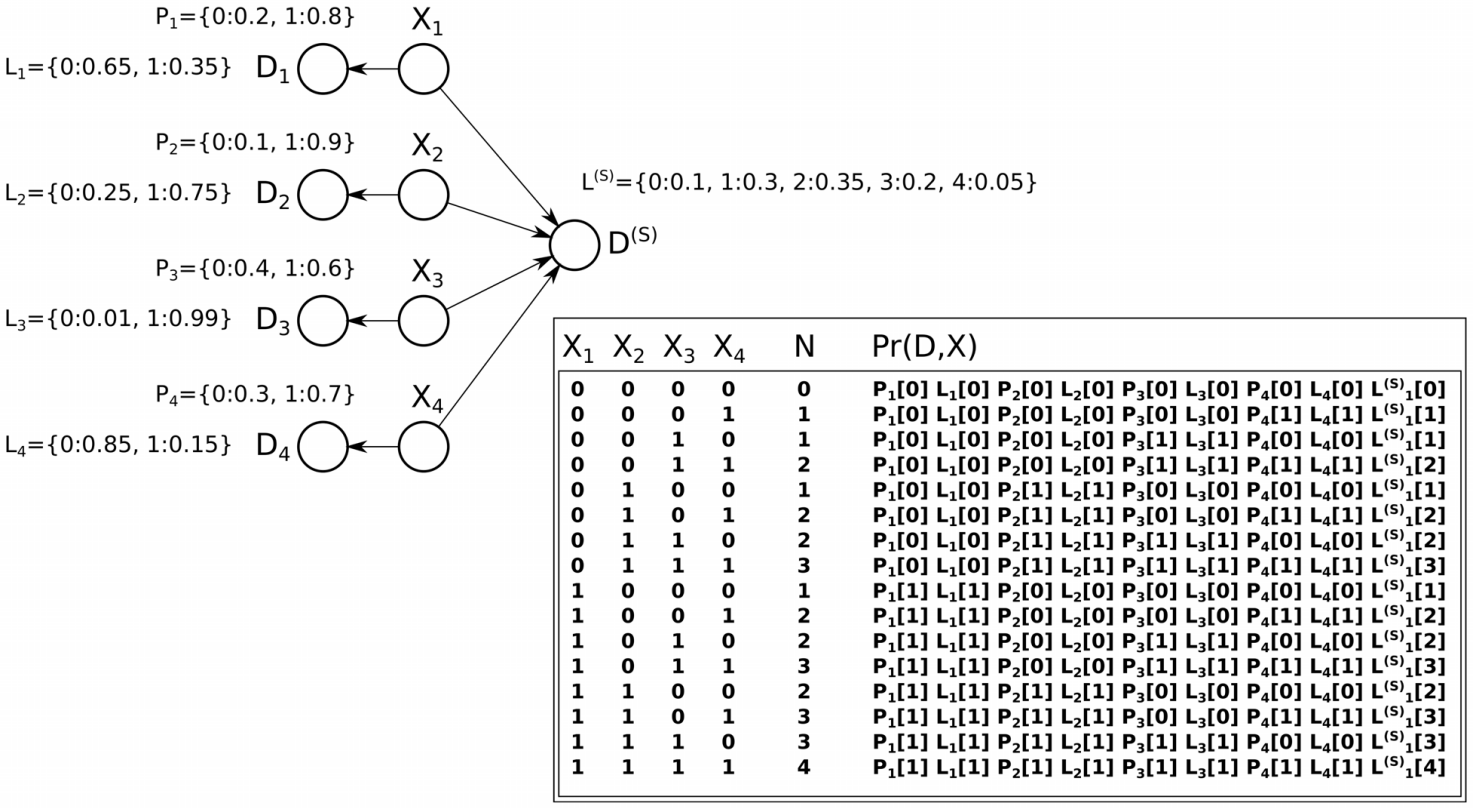
Illustration of the enumeration approach. Super-exponential enumeration is illustrated using a simple digraph. The protein prior for protein 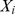 is denoted using the vector 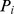 (written using Python dictionary notation), where 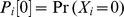 and 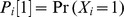. Likelihoods due to unique evidence for protein 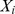 are denoted 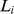, and the likelihood due to shared evidence is shown using 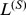, both using the same notation. The scores populating the 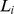 and 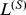 vectors comes from the peptide-level likelihoods indicating the quality of the match between the peptide and any matching spectra (*i.e.* these scores come from the conditionally independent product of PSM scores for that peptide). For example, the prior probability on protein 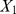 is 0.8, and a unique peptide corresponding to protein 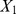 has the score 0.35 (indicating the relative likelihoods are 0.35 versus 0.65 for the respective hypotheses that the peptide matching spectrum 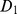 is created by protein 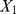 versus the hypothesis that the peptide is not created by protein 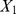). The inset shows the table produced by enumerating all distinct protein configurations, and the resulting joint probability with all data (both unique and shared). This computational cost of this enumeration is in 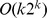 for 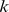 proteins.

Figure 3 illustrates this super-exponential inference algorithm on the small graph of the type shown in figure 2. Four proteins with different prior probabilities and unique spectral evidence are marginalized via brute force: for each of the 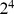 protein configurations, 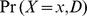 (the joint probability of the protein configuration 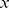 and all observed data) can be enumerated in super-exponential time (*i.e.* it takes exponential time for each protein multiplied by the number of proteins involved). The values in the column containing 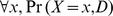 can then be used to compute marginals for a particular protein (*i.e.* protein 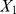). This is accomplished by separately computing the denominator and then the numerator from equation 1. The denominator can be computed by summing over all values in the column (*i.e.* 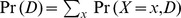), and then the numerator for the protein 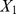 can be found by computing the sum of values in the same column, but only where 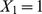 (*i.e.* 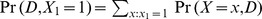). A small savings is achieved by passing over the table one time to compute the total sum, (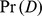 used in the denominator of equation 1), as well as the constrained sum (*i.e.* the numerator) for each protein, thereby computing each value in the column 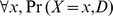 only once. Thus the cost of computing posterior probabilities for 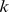 proteins is in 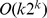 time (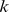 proteins 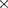 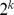 steps per protein).

#### Time and space requirements

Power-set enumeration can be performed in 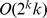 time: there are 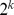 protein configurations, each of which is applied to a running total of joint probabilities for 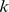 variables. Despite its inefficient runtime, power-set enumeration requires only 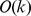 space (enumeration of the power-set can be performed by incriminating a large base-2 number, and therefore does not require *storing* the entire power-set).

### A Quadratic Dynamic Programming Algorithm for Exact Inference

**Figure 4:**
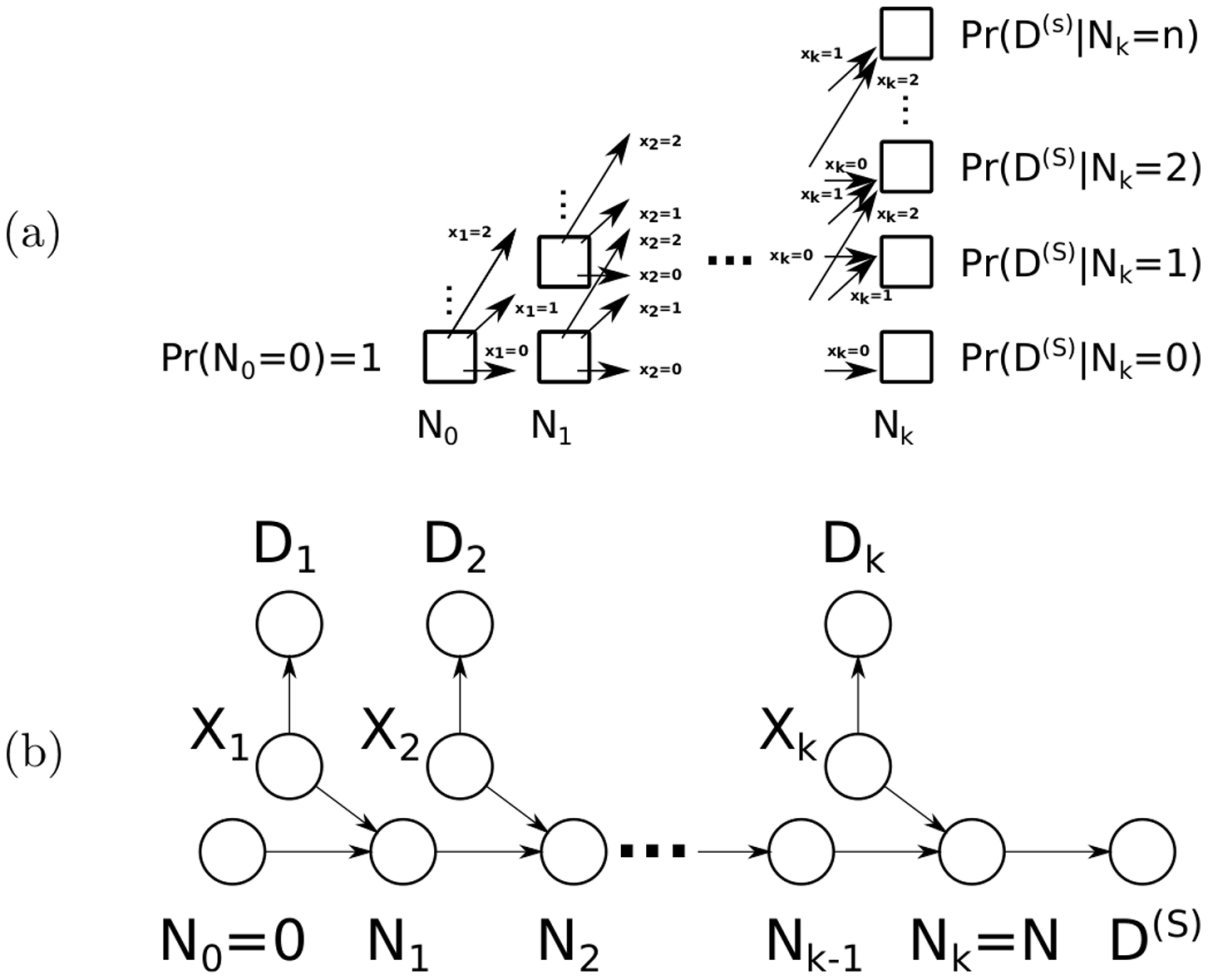
A quadratic dynamic programming approach and its generalization. (**a**) A dynamic programming approach to solving the problem from figure 2. In this approach all values of 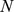, the information on which 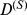 depends, are computed after successively including every next variable 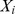. This allows paths in the exponential tree generated by the power-set to be merged when they result in the same value 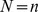, and thus allows a forward-backward algorithm to compute inference in quadratic time and space. (**b**) A general path graph can be constructed whenever the operation performed by the node 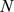, on which the shared data 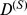 depends, can be decomposed as a series of consecutive operations that aggregate 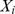 one at a time. This corresponds to operators with commutative and associative properties. The resulting transformation resembles Heckerman’s temporal transformation, which also uses quadratic time and space.

Figure 4a demonstrates the idea behind the quadratic dynamic programming algorithm: Each protein is added (with its state still a random distribution) one at a time, resulting in layers of partial sums. These partial sums work toward building the sum of the present proteins 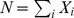. At each layer 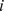 in the table, we compute the distribution on the random variable 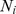 (the random variable for the partial sum defined by 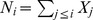). Because the variable 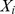 is still unknown, every possible values of 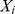 is associated with edges connecting cells in layer 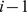 to the appropriate cell in layer 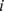. For example, the an edge labeled 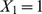 connects the 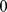 row of 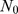 (*i.e.* the event that the random variable 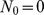) to the 1 row of 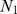 (*i.e.* the event that the random variable 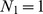), because 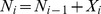. Each edge for 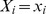 is weighted by the product between the prior that the protein has that assignment and the unique likelihood contribution that arises from that protein taking that assignment:
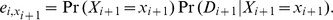



These layers are built so that the final layer is the cumulative sum of all proteins: 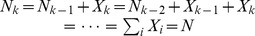. In this manner, all possible paths to arrive at every possible cumulative sum 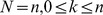 are available in the graph shown in figure 4a.

After the graph is initialized, two passes are performed: one from the left and one from the right (these passes are sometimes denoted a “forward-backward” algorithm, a special class of junction tree message passing on a path graph commonly used by hidden Markov models (HMMs). Denote row 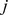 of layer 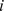 as 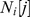, which indicates that 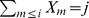. The pass from the left computes the marginal probability of arriving at this given cell through all possible weighted left-to-right paths, denoted 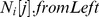 for row 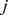 of layer 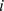. The algorithm starts with 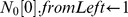, because 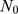 has a 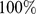 probability that it is 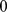 (the outcome for the layer is indicated by the row number). These values are used to fill the entire table by propagating left-to-right in the following manner:
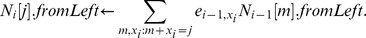



The second pass, from right to left, is performed in an almost identical manner. For each value in the table, we compute the marginal probability of arriving at this cell through all possible weighted right-to-left paths. Before starting the right-to-left pass, every node in layer 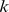 is initialized with the appropriate likelihood due to the shared data: 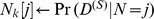. Then the same propagation is performed, but from right to left:
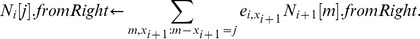



The result is that every cell in the table computes the marginal probability of all paths passing through it from left to right and from right to left (this is a standard “forward-backward” algorithm). The likelihood of being in a given node can be computed by 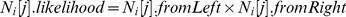. Thus, the posterior that any variable is in a certain state 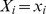 is computed by the total weight of all paths that pass through edges assigning 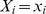:
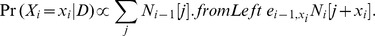



For the three preceding equations, out-of-bound indices (*e.g.* querying 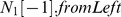 and 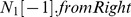) should return zero.

Note that figure 4a is drawn in a way that underscores its applicability to inference problems where each variable has more than two possible states (as indicated by more than two edges radiating out of each node, and with labels not limited to binary states).

Figure 4b generalizes this approach and merges each layer 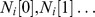 into a single variable 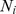. The result is a graph with a tree decomposition that is visibly simple; in fact, it closely resembles a hidden Markov model (HMM), for which the tree decomposition is trivial using a similar forward-backward algorithm [18]. This graph generalizes the dynamic programming performed in figure 4a so that it can be applied to similar problems by transforming the graph and then performing junction tree inference. The generalized algorithm is an instance of Heckerman’s transform [15], [15,17], which is sometimes referred to as a “temporal transform”.

From the generalized figure 4b, it is easy to understand the algorithm described above: in figure 4a, passing through the node 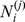 indicates that the partial sum 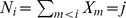. For this reason, we can see that propagation from the left accumulates the priors and unique likelihood contributions that would lead to 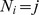.
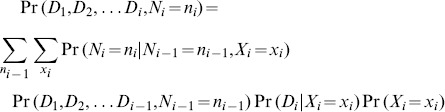



Likewise, propagation from the right computes the remaining likelihood terms, along with the shared likelihood:
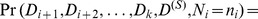





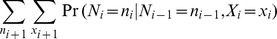





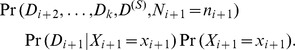



For both equations above, we exploit the fact that 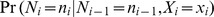 is 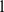 if and only if 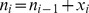 (and is otherwise 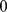). For each of the two equations above, this allows us to collapse the nested sum into a single sum over the variable with a smaller domain (if all variables 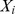 are binary, as in the case of distinguishing splice variants, then 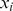 will be have a smaller domain than 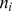).

Lastly, we see that the posterior for a protein, which is always proportional to its joint probability with the data, is computed by the forward-backward probability passing through edges labeled 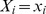:



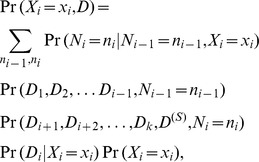



which is given by the algorithm.

#### Illustrative example of the quadratic algorithm

**Figure 5:**
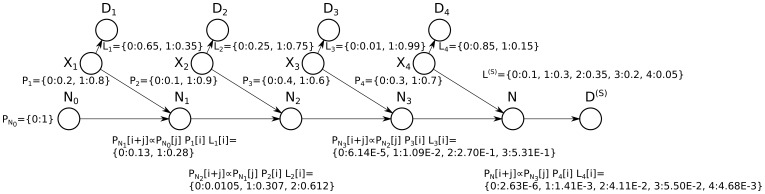
Illustration of the quadratic dynamic programming approach. The quadratic dynamic programming approach is illustrated using the digraph from figure 3: One by one, each protein is added to the initially empty total number of present proteins, represented by the random variable 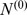. Thus, the probability distribution for each partial sum 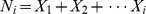 is computed and stored in the vector 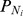. Finally, the shared evidence 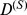 is included, as it depends exclusively on the number of present proteins 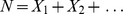. Inference for a particular protein (*e.g.* protein 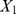) could be performed easily by performing another forward pass with the constraint that 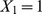, and all protein posteriors would thus be computed in cubic time with the number of proteins (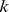 proteins 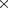 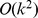 steps per protein); however, a subsequent right-to-left pass could be used to compute all protein posteriors in 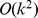 time via the forward-backward algorithm.

Figure 5 illustrates the forward pass of the quadratic inference algorithm on the small graph shown in figure 3. The posterior probability for an arbitrary protein (*e.g.*n 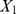) can be easily found by passing the messages left-to-right once (to compute the denominator of equation 1), and then by passing passing messages left-to-right once more with the constraint that 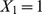 (to compute the numerator of equation 1); however, by using right-to-left pass (*i.e.* the “forward-backward” algorithm), the marginal probabilities of all proteins can be computed in roughly the same amount of time required to compute one protein posterior in this manner (*i.e.* quadratic time).

#### Time and space requirements

This approach (and, in general, Heckerman’s transform) reduces the runtime to 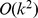, but the table used for dynamic programming uses 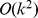 space. This time-space trade-off can be considered almost universally favorable compared to power-set enumeration: for problems when 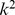 would become too large to store in the RAM of a modern computer, 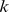 would be so large that 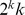 would result in an astronomically large runtime for power-set enumeration; however, space will likely be the limiting factor in applying Heckerman’s transform, and so like other 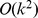 algorithms, it cannot be applied as-is to very large problems as mentioned in [19]; for completeness it should also be noted that the VE1 algorithm [19] is not well-suited to the problem in figure 2 because it is query based, meaning that it performs best when only a small subset of the variables need to be solved.

### The Probabilistic Convolution Tree: a more Efficient Dynamic Programming Algorithm

**Figure 6:**
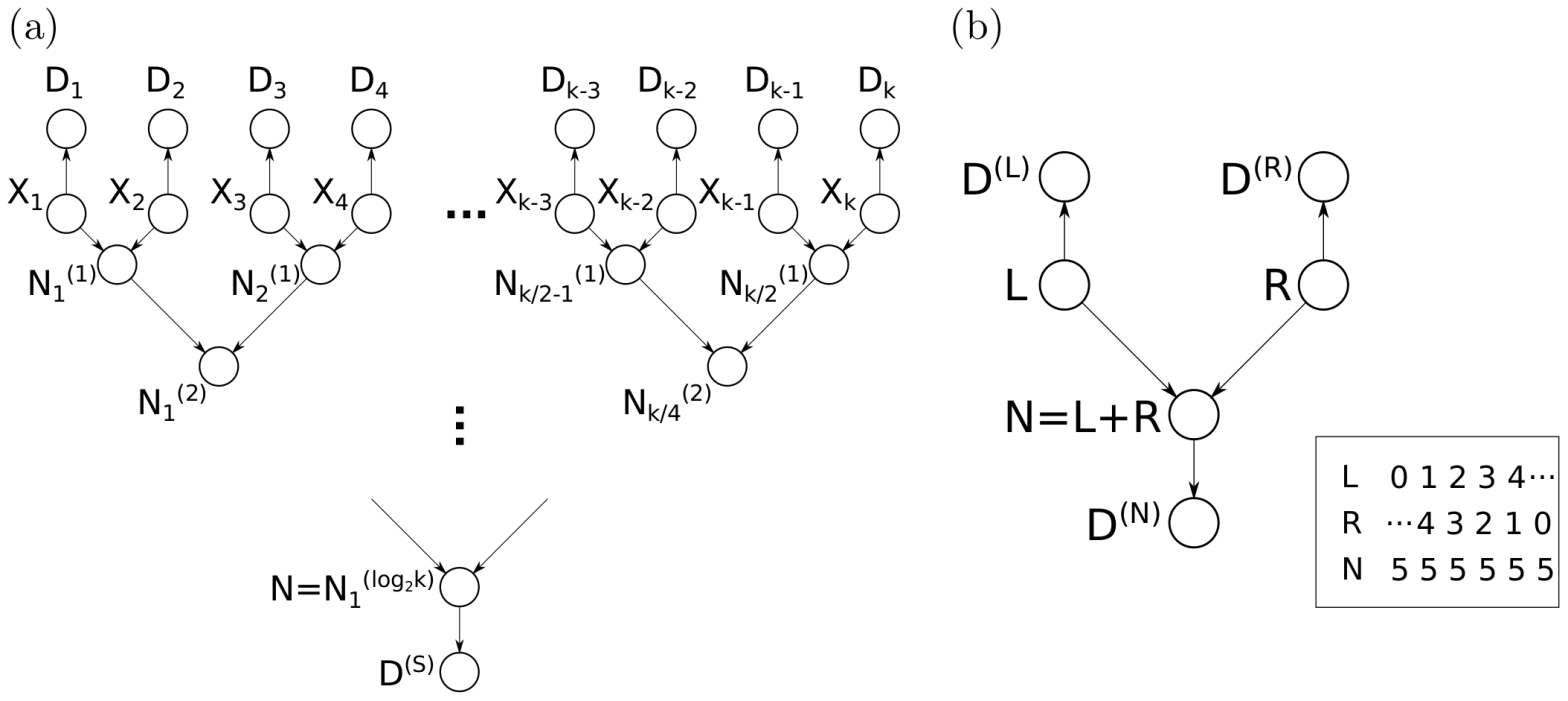
Faster dynamic programming using the convolution tree. (**a**) An alternate transformation for efficiently computing posteriors for all proteins. Instead of unrolling the commutative and associative operator one protein at a time as performed by the quadratic dynamic programming algorithm, variables are paired successively, resulting in a tree with depth 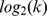 (when 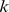 is a power of 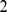). (**b**) Inference on this tree can be performed by solving a minimal ternary node structure and then proceeding inductively: all nodes (except for the proteins themselves) have two parent subtrees, 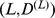 and 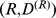, and one child. The parent subtrees connect the node of interest to all data reachable through the parents above (partitioned into 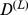 and 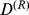, respectively), and the child subtree connects to all data reachable below, (denoted 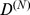). The joint probability with all data above can be passed as messages from parents to children, and the likelihoods given data below (that is, all data reachable through a downward edge out of a given node) can be passed upward from child to parents. Each of these three messages turns out to be a convolutions (shown in inset). For example, all ways that 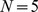 can be computed by a shifted and reflected dot product, which finds all 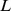 and 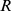 with a sum of 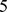. Thus the prior probability for 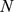 can be seen as a vector equal to the convolution of the prior probabilities of prior probabilities for 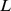 and 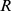. These convolutions can be performed with fast Fourier transform (FFT) in 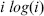 time (where 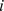 is the size of the possible state space of 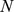). If the vectors are very sparse, then a standard discrete Fourier transform-based (DFT) convolution may be faster.

The probabilistic convolution tree, shown in figure 6 is similarly motivated to the dynamic programming shown in figure 4; however, where the quadratic dynamic programming algorithm constructs a chain 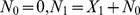, 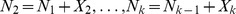, the probabilistic convolution tree proposes a divide-and-conquer approach. First we present an algorithm applicable when 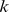 is a power of 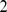, and then generalize it. For this reason, all logarithms are base 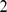: 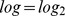.

Probabilistic adder nodes are partitioned into multiple layers: 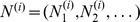, where 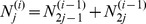. In this manner, every node in the tree from layer 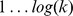 has exactly one child and two parents. Layer 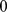 is composed of the parent-free variables 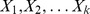; each nodes 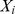 in this layer has two children: their unique data 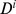 and the probabilistic adder node in layer one, 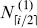, which depends on them.

Because of this consistent structure after layer 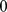, the entire tree can always be viewed from a single probabilistic adder node 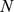 as having two parents 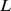 and 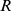 as parents. These parents, along with the data reachable through them, constitute subtrees 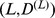 and 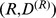. Each node has a single child subtree containing all data below 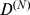 (Figure 6). 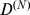 includes the shared data 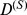 as well as any other data nodes reachable through the edge down from the node. For instance, the tree as seen from the perspective of node 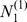 has 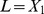, 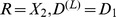, 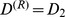, and 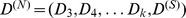. Because this ternary structure is ubiquitous in the tree, a message passing algorithm can be constructed by using only the subtree from figure 6b.

#### Example: computing prior probabilities

As an example of the motivating idea behind the probabilistic convolution tree, first observe that the prior probability of a node can be constructed easily using the prior probabilities of its parents:
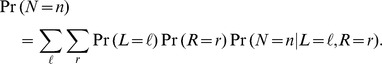



And because 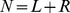, this can be condensed to a single summation:
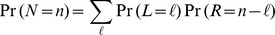



Priors are known for all nodes in layer 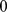, and so it is clear by induction that a prior can be computed for every node (layer 0 is the base case and every prior in the next layer can be computed given the priors from the previous layer).

However, as written above, computing the prior for every node in the tree would be at best a constant speedup over the quadratic dynamic programming approach, because the two nodes in second to last layer will have state space 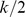 and 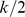, and all pairs must be combined to form the prior on the final node, 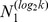 (enumerating these combinations requires quadratic time). However, we can now employ convolution at each node (see figure 6b inset), and so it can be computed in 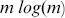 time, where 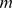 is the state space of the node 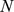 being updated. Note that when the vectors are very sparse, the convolution can be performed more efficiently using a standard discrete Fourier transform (DFT) rather than a fast Fourier transform (FFT). Also note that a vector that can easily be decomposed into a sum of dense vectors whose nonzero indices do not overlap, then Fourier transformation can be performed using a hybrid approach on the results of the decomposition. If a probabilistic statement with a single unassigned variable (*e.g.* 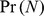, 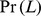, or 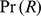) is considered as a vector (with all outcomes of that variable enumerated in order), then the summation described in the equation above is equivalent to
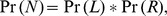
where 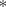 is the vector convolution operator.

We now proceed to outline the convolution tree algorithm, which operates in a similar manner as this example.

#### Step 1: passing messages down

First, the convolution tree algorithm computes the joint probability of each node with the data above it. This can be performed almost identically to computation of prior probabilities in the example above. Also, like above, the base case is known for all nodes in the layer 0 (it is simply the element-wise product between the node’s prior and its likelihood from unique data). Thus in the same manner presented in the example above, the joint probability of the node’s state with all data above, 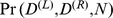, can be computed using a convolution:
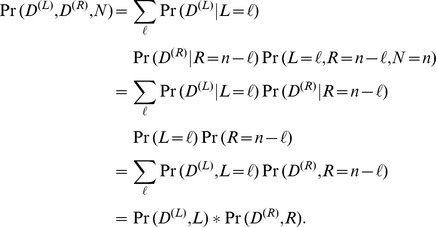



Like the example above where prior probabilities are computed, each node in layer 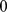 has a known joint probability with the data above. And so, proceeding layer-by-layer, for each node we compute the joint probability of that node with data above it.

#### Step 2: passing messages up

The second part of the convolution tree algorithm is to pass messages upward through the tree after all messages have been propagated downward (*i.e.* after completion of step 1). Where step 1 passes down the joint probability of each node with the data above it, step 2 computes the likelihood given all data that can be reached below: for the left parent, we compute 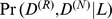 and for the right parent we compute 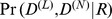. Note that we only need values proportional to these probabilities, and so they can be normalized before proceeding to the next layer in the tree for better numeric stability. These messages can be defined thus:
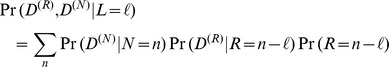


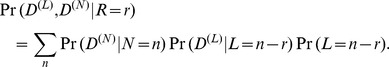



Where the forward pass (in step 1) represents addition, this backward pass represents subtraction, which presents also convolution, but because one operand is negated, its indices are reversed. Also, the result of the convolution will not start at zero, but will instead start at the minimum value achieved by the subtraction. In this case, we do not want the negative values because they are impossible in the source variable. Thus the above equations can be written as convolutions where one vector is reversed and where a slice is taken from the result (to undo the shift and remove impossible outcomes). So the final results can be computed
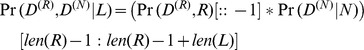





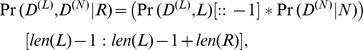
where 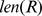 and 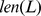 depict the number of states for 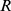 and 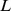, 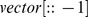 reverses the vector (*i.e.* it reflects it), and 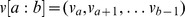 slices the vector to remove the unwanted shift mentioned in the paragraph above.

As messages were propagated downward before, these messages can be passed back up the tree to form the likelihood given data below for the left and right parent nodes. For example, for the left parent 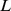, its likelihood given all data below will be initialized by the message passed back, 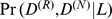. Now that the node 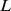 has its likelihood given the data below, it can pass messages up the tree. This process continues until all messages have been passed up to layer 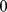, which contains the proteins themselves.

The posterior for node 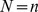 can be computed by multiplying the joint probability with data above by the likelihood given data below that node:
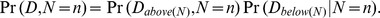



Similarly, the posterior for protein 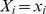 can be computed by multiplying the joint probability with data above (for each protein 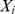, the data above consists only of its unique spectral evidence 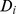) by the likelihood given data below:
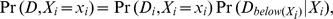
which is proportional to 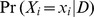. Note that when a value is proportional to the posterior probability, then the posterior proportionality can be computed by dividing by the sum:



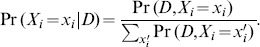
(1)Like the quadratic dynamic programming algorithm, the convolution tree algorithm described here is applicable to variables with more than two states. It also does not require that the left and right parents of a probabilistic adder node have identical state space, although of course an FFT-based convolution will be faster when this is the case, because it will not need to pad the shorter vector with zeros.

#### Illustrative example of the probabilistic convolution tree algorithm

**Figure 7:**
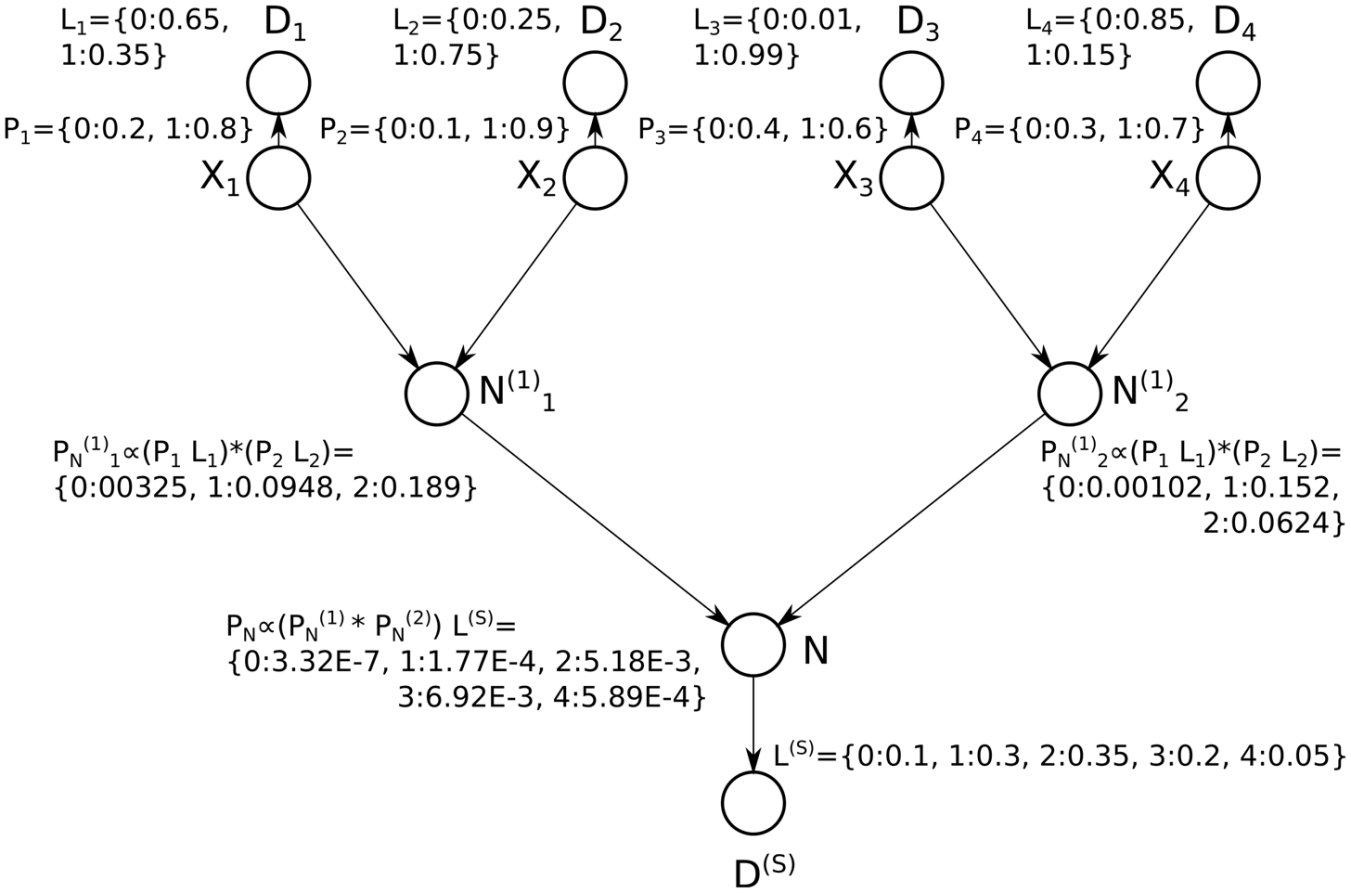
Illustration of the probabilistic convolution tree. The convolution tree is illustrated using the digraph from figure 3 and figure 5: Messages are passed down the tree (via step 1). A subsequent pass would send messages up the tree (step 2), computing the protein posteriors in sub-quadratic time. Note that the normalized vector 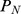 is equivalent to the distribution 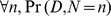, and is identical to the normalized vector of the same name computed by the quadratic algorithm illustrated in figure 5.

Figure 7 illustrates the convolution-based inference algorithm on the a small graph of the type shown in figure 2. Messages are passed down (via step 1). The posterior probability for an arbitrary protein (*e.g.*n 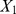) can be easily found by passing the messages down once (to compute the denominator of equation 1), and then by passing messages downward once more with the constraint that 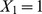 (to compute the numerator of equation 1). however, step 2, which subsequently passes messages back up the tree, reuses the shared computations for these proteins, and computes the posterior for every protein in roughly the time necessary to compute the posterior for any single protein (sub-quadratic time).

#### Time and space requirements

As implemented, the runtime of the convolution tree can be shown to be 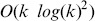, and the space requirement can be shown to be 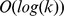. Sparse vectors can be convolved more quickly by using the discrete Fourier transform (DFT).

A fixed number of convolutions are performed at all nodes. Layer 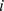 will have 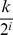 nodes, and the length of the state space for that convolution is 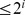. Thus the cost of FFT convolution for a node at layer 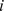 is 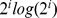. The entire runtime can then be computed easily using the expansion 
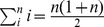
:
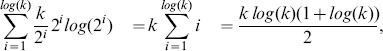
which is on the order of 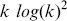.

The memory consumption is computed with a similar strategy. The simple implementation of the convolution tree method described here requires 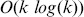 space to store the two vectors 
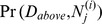
 and 
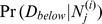
 for each node 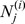 in the tree, because at layer 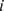, the length of the vector storing each of these is 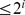, and there are 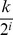 such nodes in that layer. Thus the total space requirement is
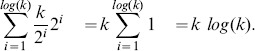



The method can be easily extended when 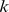 is not a power of two by simply adding dummy variables 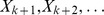 with 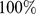 prior probability of being absent (thus they do not influence the sum) and with no unique evidence). These variables are added until the total number of variables is a power of 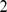. Using a 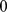 prior is important for this approach, because it prevents 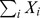, and subsequently 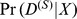, from being altered by including these dummy variables. Even though this approach is inelegant, it can be easily seen to produce the same order runtime when 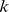 is not a power of 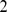, because in the worst case, 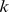 must be roughly doubled to perform inference, which would simply change the constant, but not the order of the runtime.

#### Extension to linear functions on the integers

The special case of computing posteriors where 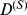 depends on 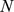 with 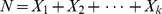 (as described in this paper), can be easily generalized to compute posteriors when 
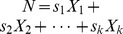
 for fixed integer scaling factors 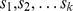. First observe (without loss of generality) that for any positive integer 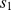, 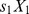 simply creates a new vector 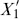 with 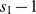 zeros padded between every entry of 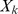:
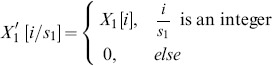



Second, observe that for any two nodes 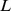 and 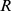, the subtraction 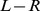 can be accomplished by reversing 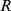 before convolution with 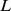 (and recording the fact that the zero index of the resulting array no longer refers to 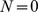, but instead refers to the minimum value achievable by the subtraction). For this reason, scaling 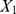 by a negative integer 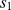 can be performed by reversing the vector 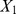 and then padding with zeros as mentioned above, and then adding normally with the convolution tree (again, in this case, each node in the convolution tree would would also keep track of the minimum integer summation value corresponding to the zero index). For completeness, when 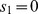 (and is thus neither positive nor negative), then the scaled vector 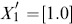, indicating a 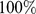 probability that 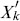 is zero; however, if the fixed value 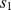 is known to be zero ahead of time, then there is effectively no edge connecting 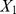 to the scaled summation 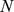, and that input can be ignored with no consequence.

Thus scaling 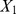 by 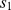 can be accomplished by simply permuting the indices of 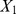 (into a potentially larger result vector): the vector 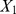 is first reversed if 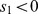 and is then padded with zeros as described above.

For simple implementation, single-input single-output scaling nodes (with input 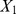 and output 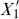 and a fixed parameter 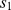) can be used to first transform any 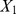 into 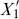, and then fed into a convolution tree node with 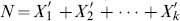. Thus we can model an integer-scaled sum without any modification to the convolution tree data structure. Messages passed backward through these scaling nodes simply undo the deterministic permutation of indices mapping 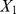 to 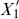.

## Discussion

### Performance on Randomly Generated Problems

**Figure 8:**
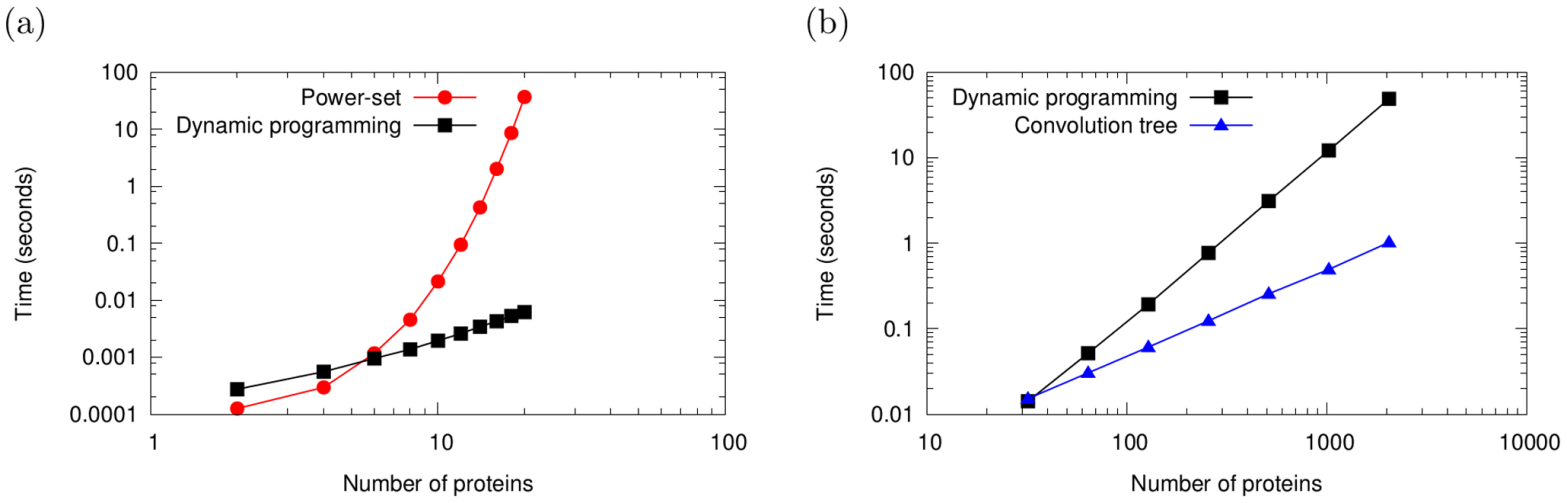
Runtime comparison between the algorithms. (**a**) A comparison of power-set enumeration and the quadratic dynamic programming approach on small problems of the form from figure 2. Note that axes are log-scaled, and so a widening gap between the curves indicates a super-linear speedup for the algorithm producing the lower curve. (**b**) A comparison of quadratic dynamic programming and the convolution tree approach on larger problems of this form. The convolution tree achieves a super-linear speedup and a super-linear reduction in memory consumption, making it applicable to much larger problems than either the quadratic dynamic programming approach or power-set enumeration. On very small problems (requiring substantially less than one second of runtime), the more sophisticated dynamic programming approaches have higher overhead, and are therefore slightly slower.

Figure 8 shows a runtime comparison between the three algorithms using a more general model, which is not restricted to noisy-or nodes and instead uses probabilistic adder nodes. Figure 8a compares power-set enumeration with the quadratic dynamic programming method on smaller problems (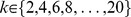). Figure 8b compares quadratic dynamic programming with the convolution tree method on larger problems (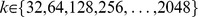). Note that both axes are log-scaled and so a growing gap between the two series represents a super-linear speedup in the runtime. On larger problems (*e.g.* 4096 proteins), the quadratic dynamic programming runs out of memory on a 4 GB computer.

Both the quadratic dynamic programming and the convolution tree have runtimes far superior to power-set enumeration. But moreover, the convolution tree offers scalability to substantially larger problems than the quadratic dynamic programming approach. For example, computing exact posteriors for 32768 proteins takes only 28.03 seconds, while the quadratic dynamic programming cannot even be run.

### Cascading Trees for more General Application

**Figure 9:**
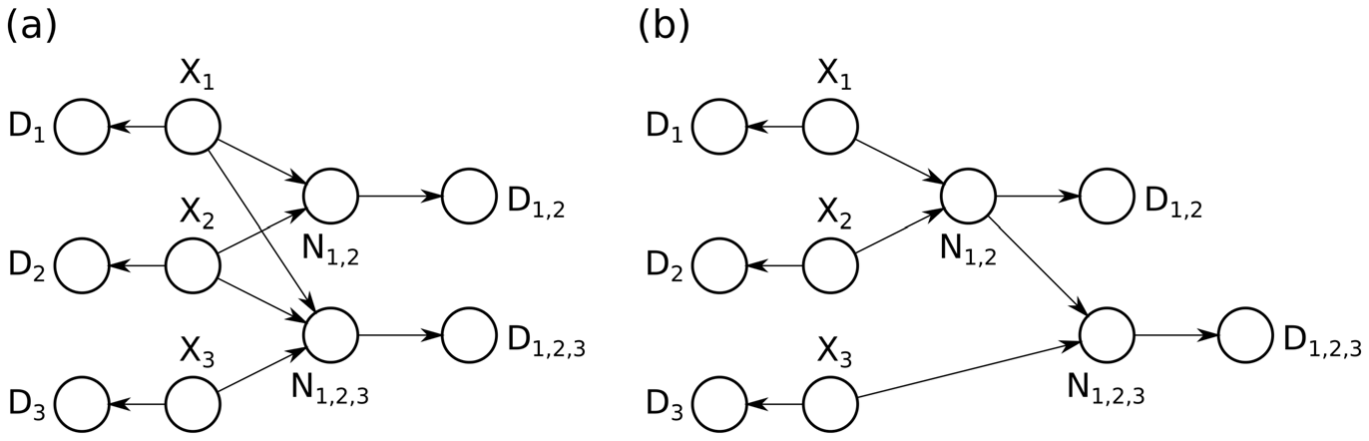
A graph and its cascaded equivalent. (**a**) A Bayesian network with probabilistic adder nodes 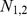 and 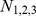. (**b**) The resulting cascaded graph of probabilistic adder nodes transforms the graph into an equivalent Bayesian network that can be solved efficiently as a convolution tree. Graphs that do not cascade into polytrees (*i.e.* graphs that have loops even after cascading nodes as shown here) can be solved with a slightly modified junction tree inference algorithm: junction tree clique nodes that consist of a single probabilistic adder node and its inputs can pass messages through convolution tree nodes (without realizing the full conditional probability distribution).

It should be noted that the convolution tree method can easily be applied when including node-specific data 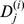, which depends only on the node 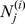 in the tree (as long as the resulting graph is still a tree): The modified method would simply multiply (element-wise) the likelihood given data below 
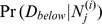
 by the unique likelihood 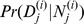 when passing messages up and multiply (element-wise) the joint probability with data above 
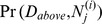
 by the unique likelihood 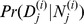 when passing messages down. This allows nearly identical runtime (point-wise multiplication is cheaper than convolution, which is already performed by the algorithm). On graphs where data is shared in a manner such that it is cascaded (*i.e.* 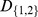 depends on the sum of 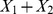, and 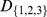 depends on the sum of 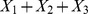, and so forth), the sums can be arranged by a simple greedy algorithm so that a probabilistic adder node 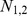 has predecessors 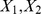 and then a second probabilistic adder node 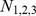 has predecessors 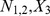 (Figure 9). Thus, cascading makes it possible to use the convolution tree even when the shared data 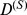 do not have *identical* predecessors as shown in figure 2. Furthermore, more general cascading can factor out shared computation so that data 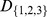, which depends on 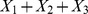 and 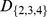, which depends on 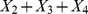 can be factored into 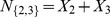 and where 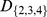 depends on 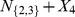 and 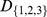 depends on 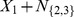. Cascading probabilistic adder nodes allows inference in the same runtime and memory usage derived in this manuscript when the cascaded nodes form a tree.

### Compatibility with Belief Propagation and Junction Tree Inference

When the graph contains loops (*i.e.* when the cascaded graph does *not* form a tree), variables can be merged into larger joint variables, and then the sum of these variables can then be fed into a convolution tree. Essentially, this demonstrates the potential to use convolution trees as specialized cliques within classic junction tree inference [8]: When inference is performed, the full joint conditional probability table would not be generated for any clique node in the junction tree with two properties: 1) The clique node contains only a probabilistic adder node and all of the probabilistic adder node’s predecessors. 2) The edges connecting the specialized clique node to other clique nodes in the junction tree would also need to carry messages of a single variable only, because the convolution tree does not allow arbitrary joint distributions of its inputs as messages passed in. 3) Messages sent along edges into the specialized clique node have disjoint variable sets (*i.e.* each variable occurs along only one message). This last criterion can always be satisfied by inserting a temporary clique node containing only the variable 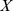 as an intermediary between the specialized clique node and any other clique nodes that send messages containing the variable 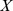.

Posterior probabilities and messages passed out of a specialized convolution tree clique node would be computed using the convolution tree, and would simply pass the likelihood of all data preceding the edge along which the message is passed (*i.e.* all data found by moving backward against the direction of message passing). For the “head” node (*i.e.* the adder), the message out would be the joint probability above. For any “input” node, the message out would be the likelihood below.

Such “intelligent” junction trees can likewise feature approximation clique nodes for use when large cliques do not meet the requirements to be specialized convolution tree clique nodes, *e.g.* clique nodes that perform mathematical approximations similar to pruning or clique nodes that use sampling methods like Monte Carlo or Markov chain Monte Carlo (MCMC). In this manner, protein inference could be performed using the benefits of three approaches: junction tree inference (which can break apart large connected graphs), convolution tree clique nodes (which can allow the junction tree to perform efficient exact inference when large probabilistic adder cliques are encountered), and approximations (available as a last resort to prevent a single remaining large clique from prohibiting inference on the entire junction tree). Approximations (which can be inaccurate and slow) would thus be avoided when exact answers can be computed efficiently. Such junction trees are examples of the recommended extensions to the collapsed Gibbs sampler mentioned in [8]. In a similar manner, pruning may be performed by first finding expensive cliques in the junction tree (*i.e.* large cliques that cannot be solved using the convolution tree) and pruning only the peptides that depend on them. This is an enhancement of the score-driven pruning algorithm defined in [13], which needed to prune *all* peptides at or below the score of the peptides responsible for computational expense.

In addition to easy compatibility with collapsed Gibbs sampling, the probabilistic convolution tree can be also be easily used with iterative approximation methods that pass messages in the original graph rather than in the junction tree: these methods include loopy belief propagation [20], variational methods [21], and expectation propagation [22]. Networks that employ a large number of probabilistic adder nodes can pass these messages very rapidly and thus arrive at an approximation very quickly, even on graphs whose tree decompositions contain large cliques, which thus do not offer significant speedup compared to brute force. Importantly, the probabilistic convolution tree can efficiently pass messages forward and backward through nodes with many predecessors, enabling these iterative approximation procedures to not longer be limited by the maximum number of predecessors from any node.

### Performance Improvement for Junction Tree Inference on a High-coverage HeLa Sample

As stated above, junction tree inference allows the belief propagation algorithm to be applied to generalized from trees to graphs by first performing a tree decomposition of a graph and then passing messages through the tree decomposition. Here we show the runtime speedup introduced by using probabilistic convolution trees within the message passing step of junction tree inference.

**Figure 10:**
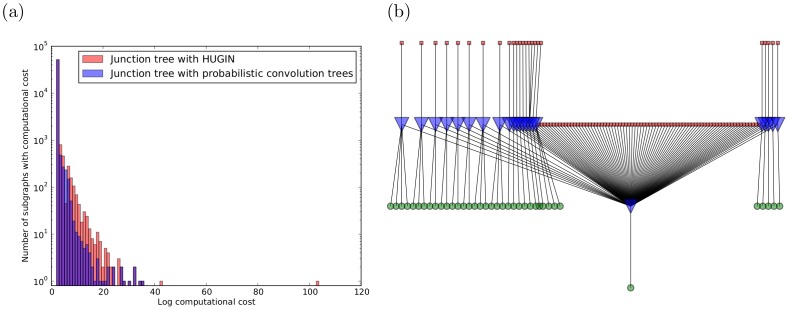
Runtime benefit of convolution tree-based junction tree over HUGIN-based junction tree on HeLa data. (**a**) Distribution of log runtimes for different connected subgraphs (24 fractions). A HUGIN-based junction tree implementation is compared to a probabilistic convolution tree-based junction tree implementation. The cost of inference will be dominated by a few outlier graphs, which do not decompose effectively using the junction tree. As a result, some connected subgraphs would require an impractical number of steps when using the HUGIN algorithm. This runtime can be improved by using probabilistic convolution trees, while still achieving the exact result. (**b**) One difficult connected subgraph from panel (a). Proteins are shown as red squares, probabilistic adders are shown as blue inverted triangles (these are the nodes can make use of probabilistic convolution trees), spectral evidence is shown as green circles. This subgraph would require 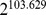 steps using the HUGIN junction tree. In contrast, the probabilistic convolution can solve this same subgraph in 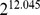 steps (and achieve an exact result).

Figure 10 demonstrates the practical benefit of using a junction tree that utilizes probabilistic convolution trees to perform belief propagation. This is demonstrated using a high-coverage (24 fractions) HeLa SILAC data set [23]. Proteins were digested with trypsin and searched against the highly redundant International Protein Index (IPI) database (using a precursor mass tolerance of 10 ppm and a fragment mass tolerance of 0.6 Da). Identified spectra were then processed with Percolator using a maximum delta Cn of 0.05.

Protein identification was performed using FidoCT (Fido with convolution trees) in the alpha release of Proteome Discoverer 2.0. Low-scoring PSMs (those with score <1%) were pruned to reduce graph connectivity as described in [13]. The resulting graph was factorized into separate connected subgraphs, and each connected subgraph was processed using two variants of exact junction tree inference: first, the state-of-the-art HUGIN [24] junction tree algorithm and second a novel junction tree approach that passes messages by using probabilistic convolution trees. The HUGIN junction tree algorithm, as described for mass spectrometry-based proteomics in [8], performs tree decomposition by merging variables into cliques (illustrated in figure 1), and then performs message passing between these clique nodes. Note that the HUGIN junction tree inference cliques require time and space that grows super-exponentially with the size of (*i.e.* the number of variables contained in) the clique. Thus the size of the largest clique dominates the computational cost; however, when probabilistic convolution trees are used, cliques formed by probabilistic adders have sub-quadratic rather than super-exponential time.

Figure 10a shows the distribution of (log-scaled) runtimes for each connected subgraph when using a standard HUGIN algorithm versus using the probabilistic convolution tree-based approach. One particular connected subgraph requires 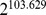 steps using the HUGIN junction tree, but requires only 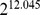 steps when using probabilistic convolution trees within the junction tree inference algorithm. This connected subgraph is depicted in figure 10b; even though its treewidth is high, exact inference can be easily performed using probabilistic convolution trees.

### Potential Impact of Convolution Trees on Protein Inference

In proteomics, the convolution tree could make it feasible to query protein databases with much greater sequence similarity than is currently possible, due to the large number of shared dependencies introduced (as shown in figure 10). Moreover, the convolution tree could be used to iteratively perform protein inference and model peptide detectability, because it can offer substantially better runtimes on large or highly complex data sets; on such data sets, iterative numeric methods (*e.g.* ProteinProphet [25]) have been demonstrated to be unstable [2], [13], [26], heuristics and human intuition can break down [27], approximations (such as pruning peptides with nonzero scores) may be forced to yield inaccurate results [3], and sampling on its own (without exploitation of 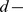
separation and mathematical properties like those introduced in this paper) cannot yield accurate probabilities in a feasible amount of time [2], [3], [8].

The convolution tree can also be used to efficiently place arbitrary categorical priors on the number of present variables or on the sum of variables. Without this advance, such priors would not be considered because they are too inefficient for large data sets: by creating a dependency between all proteins, such a prior would render factorization impossible. Without factorization, even a runtime quadratic in the number of variables (*e.g.* using the quadratic dynamic programming approach) could potentially become the factor limiting efficiency (not to mention the limitations of the quadratic space requirement). A sub-quadratic method with low space complexity could be used to bring the applicability of such priors to many graphical inference problems.

### Application to Probabilistic Generating Functions for Partition Combinatorics and Linear Diophantine Equations

The convolution tree can also be trivially applied to classic partition problems from combinatorics [28]. Given some target value 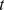 and elements 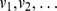, compute the total number of distinct integer tuples 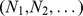 that satisfy 
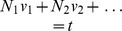
. Traditionally, generating functions have been used to great success for such problems; however, converting the generating function solution to the desired result involves computing a particular polynomial coefficient from generating function, and can be time-consuming. Furthermore, the probabilistic convolution tree framework relaxes the strict equality required by generating function variants: the probabilistic convolution tree would not require the sum be *exactly* the target value. Instead, it would be compatible with an arbitrary likelihood function that weights values by the quality of the approximation between the target 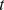 and the sum 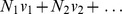. In practice, this problem can be used to compute the total number of unique ways to make change for a given amount of money, and can also be applied to deciding if an observed target mass 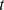 can be made from specified set of elements (or, more generally, components) with respective masses 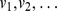 (and to compute the marginal distribution on the quantity of each element). A generalized two-dimensional variant could also constrain the total valence of the elements employed used.

Likewise, the probabilistic convolution tree can be applied to the highly related problem of linear Diophantine equations, which seek integers 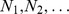 such that 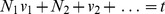 for some target value 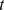. The probabilistic convolution tree can decide if the equation is feasible (using integers 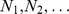), as well as to compute marginal probabilities for each integer coefficient 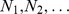. Although the problem of finding *whether* such an integer solution exists is known to be polynomial, the full problem of computing a joint solution in the integers 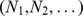 is NP-complete [29].

### Generalization to Multiple Dimensions and Applicability to Knapsack Problems

The convolution tree trivially generalizes to multidimensional problems where there is an additive behavior in all dimensions. For instance, as mentioned above when mentioning the applicability to elemental decomposition, we can consider all variables 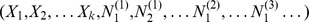 to have two dimensions. In the case of elemental decomposition, each variable can store a two-dimensional random distribution as a matrix where the row index maps to the discretized mass, the column maps to the discretized valence, and the value in the matrix cell at row 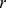 and column 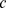 is the joint probability that the variable simultaneously has the mass corresponding to 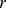 and the valence corresponding to 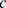. Note that the algorithm is identical with two exceptions: First, when adding two parent variables 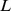 and 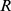 during a forward pass (step 1), the result will now be computed via a two-dimensional convolution. Second, after two-dimensional convlution is performed during the backward pass (in step 2), a contiguous submatrix is retrieved via a two-dimensional slice (rather than a one-dimensional slice).

As is the case for the univariate algorithm, the resulting convolutions can be performed using two alternative approaches: First, in the case when matrices are sparse, convolution can be performed efficiently using direct convolution when the joint distribution of 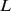 and the joint distribution of 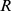. Alternatively, when the matrices representing these two-dimensional distributions are dense this two-dimensional convolution can be performed by using more sophisticated approaches [30]. The algorithm is the same in any number of dimensions as long as multidimensional convolution is used throughout, and as long multidimensional slices are taken in step 2. In this manner, it can be thought of as a polynomial time approximation scheme (PTAS) for a probabilistic generalization of the NP-hard knapsack problems. Likewise, it can efficiently solve other combinatorics problems (where counting is used probabilistically or non-probabilistically). The multidimensional variant can also be used to merge variables and remove loops from cascaded graphs that are not trees.

### Application to using Truncated Sums in Forward Error Correction

Because the convolution tree method can be used with any cascaded graph of probabilistic adder nodes, then it can be applied to myriad other problems. One simple example is the extension of probabilistic forward error correcting codes to efficiently utilize a greater variety of error correcting information. Using the notation from [31] the unique data 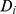 would correspond to 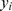, and a subset of those probabilistic adder nodes in the tree could influence node-specific 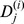, which would allow inclusion of data that depends on the sum of nodes above, or optionally, a truncated sum, from which a probabilistic adder node can compute multi-bit summary statistics about the bit string (parity is an example of such a single-bit summary statistic). For certain coding schemes that use moderate block sizes, this method could be used to infer an optimal (*i.e. maximum a posteriori*) estimate for the input binary bitstring, as well as probabilistic confidence estimates for each bit in the bitstring. Turbo decoding and low-density parity-check codes (LDPC), which are both popular inference methods for forward error correction, have been shown to be instances of Pearl’s loopy belief propagation [31], [32] (loopy belief propagation is described above). Because convolution trees can be used within belief propagation (see “Compatibility with belief propagation and junction tree inference” above), they can also be used with loopy belief propagation to perform more efficient message passing of probabilistic information related to cardinality, parity, sums, and other probabilistic adder structures that cannot be efficiently accomplished with noisy-or nodes. And because the operations performed in the probabilistic convolution tree are basic digital signal processing operations (FFT, element-wise product, etc.), and thus could potentially be implemented efficiently as an integrated circuit.

### Application to Probabilistic Demixing of Chimeric Mass Spectra

**Figure 11:**
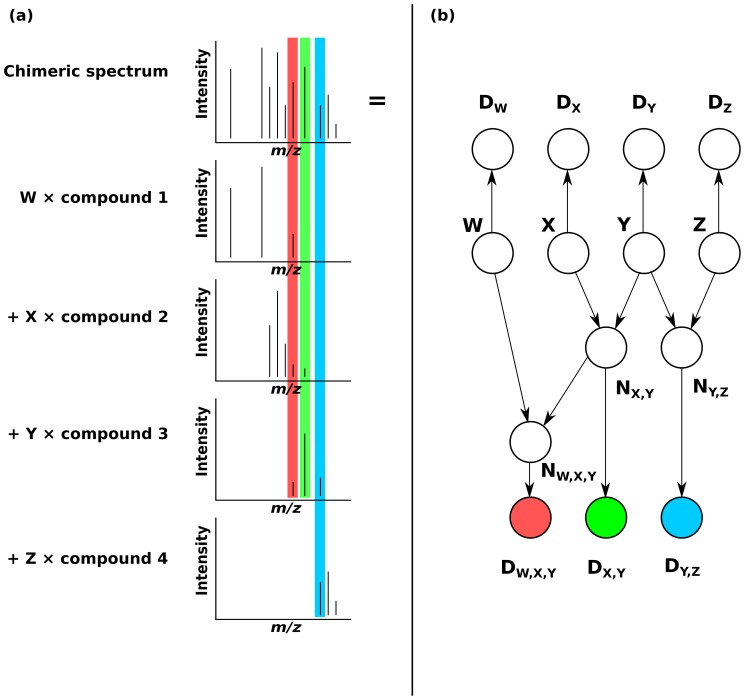
Decomposition of a spectrum into its constituent compounds. (**a**) A probabilistic demixing (a problem highly related to deconvolution) problem from mass spectrometry. An observed chimeric spectrum with data 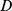 is composed of a linear combination of four different compounds and with unknown relative abundances 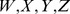, which we want to infer. Three 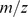 values that can receive contributions from multiple compounds are labeled with the background colors red, green, and blue. (**b**) The resulting cascaded graph of probabilistic adder nodes. The variables 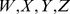 are discretized into relative abundances of interest. Conditional probabilities individually treat each intensity as proportional to the abundance of the compound that produces it. Data unique to each compound are labeled 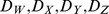, and are conditionally independent given 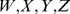. Shared evidence nodes are colored to correspond to the background colors from (a). Probabilistic adder nodes are cascaded to build a tree for probabilistic inference, enabling the computation of a posterior distribution for the relative abundance of each compound.

The convolution tree can also be applied to demixing problems. Figure 11 depicts a classic example from mass spectrometry: four compounds of unknown relative abundance contribute to a chimeric spectrum. By discretizing the possible relative abundances (in the future it also may be possible to extend some of the ideas presented in this manuscript to continuous problems), probabilistic adder nodes can be cascaded to compute posterior probability distributions on the relative abundances of each compound without jointly enumerating the four-dimensional space of all possible relative abundances. This graph could even be augmented with an arbitrary prior on the number of compounds present. A simplification would threshold peaks in the chimeric spectrum into two categories (“intense” and “not intense”) and then perform inference using a convolution tree whose base variables are binary, similar to the formulation for protein inference. Regardless of whether binary variables or binned continuous variables are used, an arbitrary likelihood model could then be used to evaluate the match between the observed peak (observed from the actual data) and the latent abundance variable for that peak (note that these likelihood functions can be peak-specific).

### Availability

The python script demonstrating the power-set enumeration, quadratic dynamic programming, and convolution tree approaches is available at https://bitbucket.org/orserang/convolutiontree.
